# Mediation of Short and Longer Term Effects of an Intervention Program to Enhance Resilience in Immigrants from Mainland China to Hong Kong

**DOI:** 10.3389/fpsyg.2015.01769

**Published:** 2015-11-27

**Authors:** Nancy X. Yu, T. H. Lam, Iris K. F. Liu, Sunita M. Stewart

**Affiliations:** ^1^Department of Applied Social Sciences, City University of Hong KongHong Kong, Hong Kong; ^2^School of Public Health, The University of Hong KongHong Kong, Hong Kong; ^3^International Social Service Hong Kong BranchHong Kong, Hong Kong; ^4^Department of Psychiatry, University of Texas Southwestern Medical CenterDallas, TX, USA

**Keywords:** resilience, randomized controlled trial, intervention, mediation, Chinese, immigrants

## Abstract

Few clinical trials report on the active intervention components that result in outcome changes, although this is relevant to further improving efficacy and adapting effective programs to other populations. This paper presents follow-up analyses of a randomized controlled trial to enhance adaptation by increasing knowledge and personal resilience in two separate brief interventions with immigrants from Mainland China to Hong Kong (Yu et al., 2014b). The present paper extends our previous one by reporting on the longer term effect of the interventions on personal resilience, and examining whether the Resilience intervention worked as designed to enhance personal resilience. The four-session intervention targeted at self-efficacy, positive thinking, altruism, and goal setting. In this randomized controlled trial, 220 immigrants were randomly allocated to three arms: Resilience, Information (an active control arm), and Control arms. Participants completed measures of the four active components (self-efficacy, positive thinking, altruism, and goal setting) at baseline and immediately after the intervention. Personal resilience was assessed at baseline, post-intervention, and 3- and 6-month follow-ups. The results showed that the Resilience arm had greater increases in the four active components post-intervention. Changes in each of the four active components at the post-intervention assessment mediated enhanced personal resilience at the 3-month follow-up in the Resilience arm. Changes in self-efficacy and goal setting showed the largest effect size, and altruism showed the smallest. The arm effects of the Resilience intervention on enhanced personal resilience at the 6-month follow-up were mediated by increases of personal resilience post-intervention (Resilience vs. Control) and at the 3-month follow-up (Resilience vs. Information). These findings showed that these four active components were all mediators in this Resilience intervention. Our results of the effects of short term increases in personal resilience on longer term increase in personal resilience in some models suggest how changes in intervention outcomes might persist over time.

## Introduction

The identification of mechanisms of change or the processes that are responsible for the effectiveness of an intervention is crucial in the evolvement of evidence-based interventions (Kendall and Terry, 2009). However, the design that are used in most outcome studies frequently do not allow for the elucidation of such mechanisms (Kazdin and Nock, 2003; Maric et al., 2012). This paper presents a follow-up to our earlier publication that described the effectiveness of a four-session Resilience intervention to enhance adaptation in Mainland Chinese immigrants to Hong Kong (Yu et al., 2014b). Our previous publication reported a randomized controlled trial to enhance adaptation by increasing knowledge and personal resilience in two separate brief interventions with immigrants from Mainland China to Hong Kong (Yu et al., 2014b). In addition to extending the previous findings to report on the longer term effects of the interventions on the secondary outcome of personal resilience, the current analyses examined a mediation model and sustained effects to explain how changes in personal resilience occurred over time. This study will focus on two questions: (1) Were the changes that occurred in personal resilience at the end of the intervention and at follow-ups the result of the components we specified? In other words, did the intervention work because of the way it was designed to work? (2) Did changes in personal resilience following the intervention influence levels of personal resilience later in time?

The need for studies that not only demonstrate that specific treatments work, but also elucidate why they work, was raised two decades ago (Shirk and Russell, 1996), and has been frequently reiterated. Nevertheless, it has recently been noted that few outcome studies are appropriately designed to clarify the processes by which change occurs (Maric et al., 2012). The information about why the interventions work not only provides insight into the mechanisms to explain outcome changes, but also points to directions for further improvement by allowing determination of the most effective components. This information may be particularly important in work with culturally diverse groups where the applicability of mediators to positive outcomes cannot be taken for granted. Mediation analyses of treatment outcomes can contribute considerably to later generations of the interventions by providing a path to enhance treatment effects and reduce costs by eliminating those components that are ineffective (Kazdin and Nock, 2003; Maric et al., 2012). The need for this knowledge has increased as programs for resilience enhancement proliferate, without parallel information about the strategies that are related to specific mediators.

Two considerations from the literature on mediation are relevant to this study. First, mediation can be tested in models (MacKinnon et al., 2002; Hagan et al., 2012; Berry et al., 2014). In this kind of model, two (or more) sets of mediators are purported to lie between the intervention and the outcome. By testing such a model, more information can become available on the processes that may be more distal to an outcome, but also be more amenable to change. So, for example, some processes such as resilience, may be shown to be a protective factor for an outcome of interest. However, in order to change resilience (the mediator closest to the outcome), the intervention program has to target some specific processes, such as self-efficacy (a more distal mediator to the outcome). If these components are also measured, the investigator has the opportunity to examine whether change in the mediator is effected as a result of changes in these components. Second, (Kazdin, 2007), among others, encouraged measurement of multiple mediators. If several components specified in the change model are examined simultaneously, the information obtained may guide program development. If, for example, both self-efficacy and positive thinking are targeted as mediators to changes in personal resilience, changes in one but not the other may mediate change in the outcome. Such findings could lead the investigators to information relevant to enhancing program effects, or selecting intervention strategies and components to retain, and those to eliminate.

An additional set of analyses in the current study was guided by frameworks relevant to the sustainability of changes, and the bases for longer term changes in outcomes (see, for example, Cohen, 1988; Maisto et al., 2008; Cohn and Fredrickson, 2010). An outcome measured at the initial stage motivates sustained changes of the same outcome over time (for example, improvement in depression immediately following the intervention predicts improvement in depression at the 6-month follow-up), thereby elucidating a potential mechanism for persistent outcome changes. We were interested in whether such sustained effects might be applied to understanding changes in personal resilience beyond the 3-month outcomes reported in our previous paper. Specifically, is there evidence for a sustainable process such that changes more proximal to the intervention in personal resilience are correlated with longer term levels of personal resilience? These sustained effects support the concept that an effective intervention creates not only an immediate short term lift, but alters underlying processes that continue to promote positive outcomes down the road.

We designed an intervention to improve the adaptation of Mainland Chinese immigrants to Hong Kong, a high-risk group who present with adaptation and integration difficulties (Yu et al., 2014b). Preliminary investigations, supplemented by the literature, led us to mediators that we proposed were necessary for successful adaptation. The first was tangible knowledge about Hong Kong institutions (including schools, medical care, housing, and transportation) which we aimed to increase in two control arms. The second, and the focus of the analyses presented here, was designed to enhance personal resilience. The Resilience intervention program consisted of four sessions, each targeting one of four processes which we hypothesized to contribute to resilience: self-efficacy, positive thinking, altruism and goal-setting. We derived these four active components from the literature on resilience (Masten, 2001; Bonanno, 2004; Greenberg, 2006; Kumpfer and Summerhays, 2006), protective factors that enhance adaptation in immigrants (Christopher, 2000; Lee et al., 2008; Wong, 2008), and information from in-depth interviews with recent and long-standing immigrants, and the social workers who had served them. We identified personal resilience as the mediator most proximal to the outcome of adaptation in our previous model (Yu et al., 2014b).

**Figure 1:**
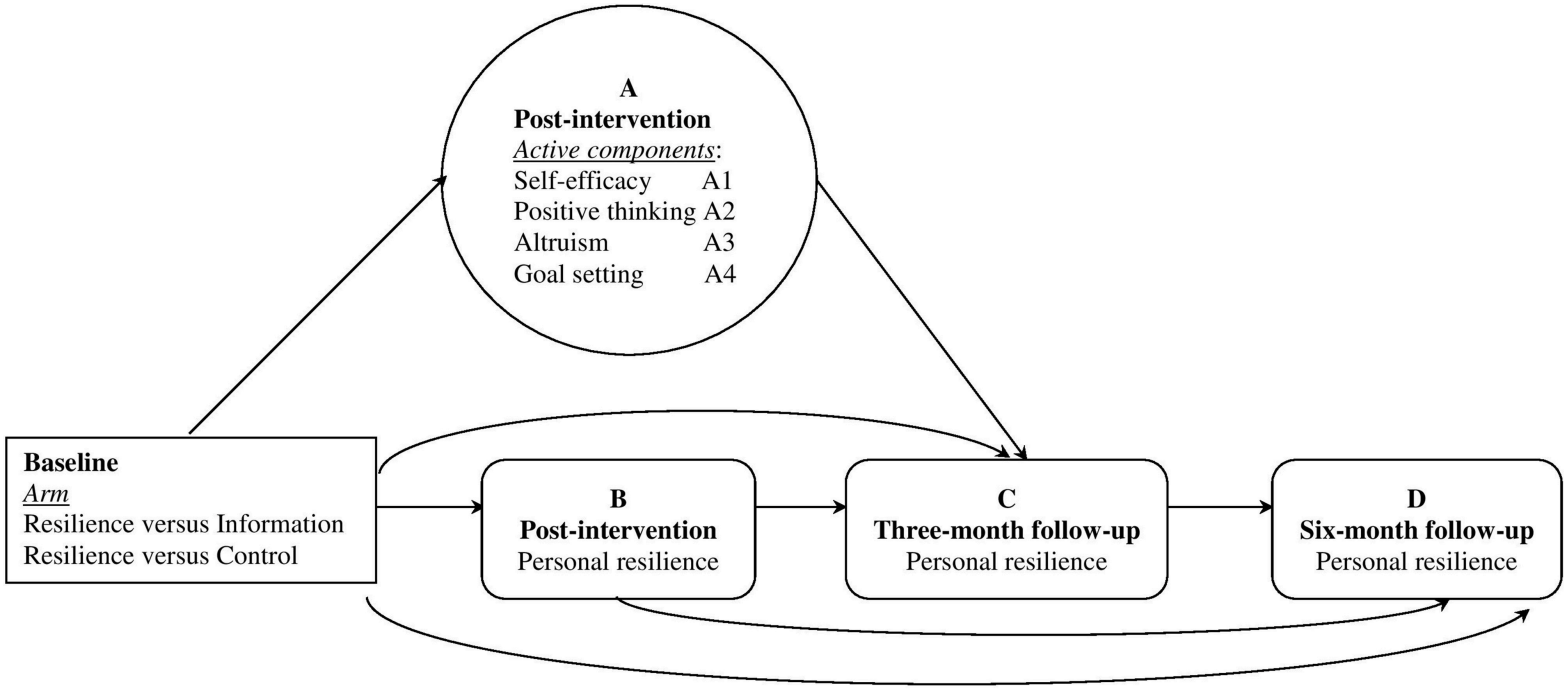
**Hypothetical models of mediation pathways of interventions effects on short- and longer-term outcome of resilience**. Separate tests were conducted for each mediator (Hagan et al., 2012). The specific analyses included: in Hypothesis 2, intervention → active components **(A)** → outcome **(C)**; and in Hypothesis 3, intervention → mediator **(B)** → outcome **(C)**, intervention → mediator **(B)** → outcome **(D)**, and intervention → mediator **(C)** → outcome **(D)**.

The assumption in the present study was that by increasing active components of self-efficacy, positive thinking, altruism and goal-setting, we would enhance personal resilience, and that following the intervention, changes in personal resilience would mediate further changes temporally distant from the completion of the intervention. This paper includes two parts: (1) to examine mediation effects on personal resilience by the active components, and (2) to test sustained effects of short term changes on long term changes. The trial compared the effects of a Resilience intervention to that of two control arms: a didactic Information intervention, and a bibliography Control where participants were given written information on the services available. We had the following aims and hypotheses:

1. We examined changes in the four active components around which we had built the four sessions: self-efficacy, positive thinking, altruism, and goal setting. We hypothesized that changes in these components would be evident for the Resilience arm but not for the two control arms. The intervention effects on these active components have not been examined in our previous report, which focused on the interventions effects on outcomes of adaptation difficulties and personal resilience in short term.
2. We examined whether each of the components acted as mediators of the effect of the intervention on personal resilience. We hypothesized that each of the four active components (A) would mediate increases in personal resilience at the 3-month follow-up (C) in the Resilience vs. the Information arm, and the Resilience vs. the Control arm (depicted in Figure 1). The four components closely correspond to our four sessions. We had no basis to predict whether some components would be more likely to show significant mediation effects than others because we were unable to find similar interventions in the literature.
3. We also investigated the sustained effects of short term outcomes on the longer term outcome. We hypothesized that increases in post-intervention personal resilience (B) would mediate the arm effect of the Resilience intervention of enhanced personal resilience at the 3-month (C) and 6-month (D) follow-ups, and increases of personal resilience at the 3-month follow-up (C) would mediate the intervention effect of enhanced personal resilience at the 6-month follow-up (D) (depicted in Figure 1).

## Materials and methods

This study was funded by the Hong Kong Jockey Club FAMILY Project (23) and was conducted in collaboration with the International Social Service, Hong Kong Branch, a non-governmental organization (NGO) that provides services in areas where new immigrants are concentrated. This study was carried out in accordance with the recommendations of ICH GCP guidelines, the Institutional Review Board of The University of Hong Kong with written informed consent from all subjects. The reader is referred to our previous study (Yu et al., 2014b) which provides more information about the community based participatory research framework utilized, and our efforts to make this program culturally appropriate, relevant and acceptable to the participants.

**Figure 2:**
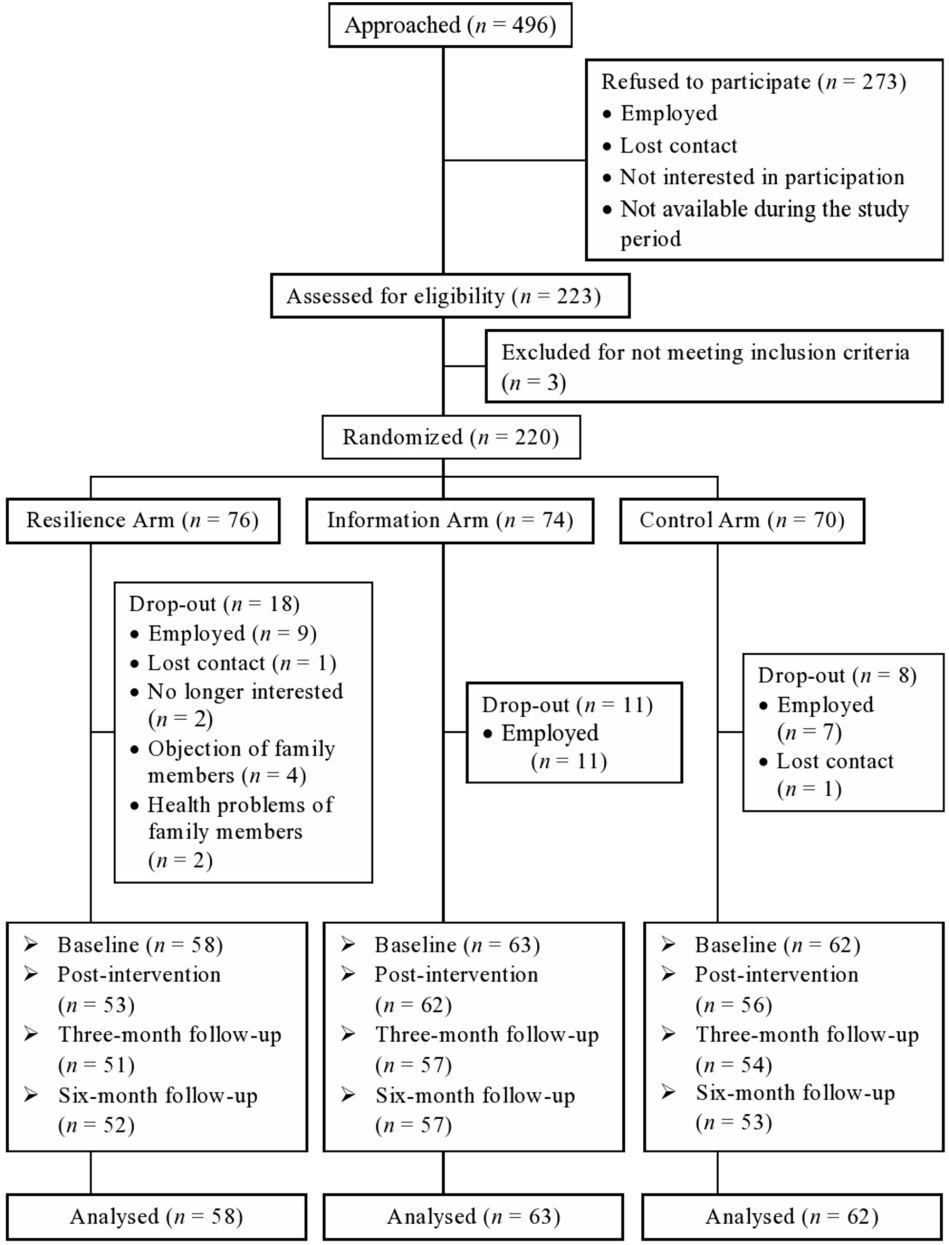
**The consolidated standards of reporting trial (CONSORT) flowchart to track participants through randomized controlled trial**.

New immigrants who had no psychiatric problems and whose child(ren) had no psychiatric problems were recruited to join the trial. In this randomized controlled trial, 220 participants were randomly allocated to one of the three arms, i.e., the Resilience arm, the Information arm, or the Control arm. Our power analyses were driven by our expectation of a change with the Resilience intervention, compared to controls, of a medium effect size (Cohen's *f* = 0.25), and our estimation of a correlation of 0.50 between repeated measures of personal resilience. These assumptions indicated that a sample size of 34 per arm (102 in total) was needed (alpha = 0.05, power = 0.80, G*Power). Our sample size estimates were based on the conservative anticipation that immigrants are difficult to recruit and retain. A previous study with ethnic minority participants showed that 20% of them did not attend a single session and 37% only completed half of the intervention sessions (Coatsworth et al., 2006). Our community partners confirmed that immigrants in Hong Kong are highly mobile and do not typically take advantage of social service programs. Estimating a drop-out rate of 40% at the 6-month follow-up, we needed to recruit 57 per arm or 171 in total. Enrollment, randomization, and the CONSORT flowchart have been described in Figure 2. Participants completed assessment questionnaires at four time points: at baseline, immediately after the intervention (post-intervention), and at 3-, and 6-month follow-ups after completion of the intervention.

To enhance retention rate after immigrant participants enrolled in the program, we used various strategies (e.g., reminder calls, make-up calls, postcards between follow-up assessments). The drop-out rate was 31.6, 22.9, and 24.2%, respectively, for the three arms, significantly lower than the estimated drop-out rate of 40%. Thus, this culturally-appropriate intervention program had better success at engaging participants than typically reported in the literature.

### Resilience arm

There were four weekly sessions, each lasting for 2.5 hours. Four session topics were extracted from the literature on positive psychology interventions (Greenberg, 2006; Kumpfer and Summerhays, 2006; Moskowitz et al., 2012; Yu et al., 2014a). The first session was about self-efficacy. Participants shared common difficulties after immigration and were introduced to the concept of resilience. Participants were asked to reflect on past successful experiences in the face of challenges. They appreciated one another's strengths and reviewed their own in adapting to Hong Kong. The second session was about positive thinking. Participants raised their awareness of the prevailing tendency in the face of difficulties to focus on the negative side and used maladaptive coping (e.g., complaints, remorse). This session targeted irrational beliefs and modeled positive reframing of existing difficulties, thereby promoting positive affect. The third session focused on altruism. Participants volunteered at a homeless center, to trigger gratitude by using downward comparison (Vollhardt and Staub, 2011) and helping those less fortunate. The fourth session was about goal setting. This component aimed to help participants identify and achieve their goals by outlining realistic pathways to those end points. Participants set goals, shared solutions to potential hardships, explicitly discussed the steps to accomplish the goals, and scheduled these steps. For example, participants who wanted a job (for example, as waitress or cashier) planned to receive job training available at the NGOs, conducted mock interviews, searched for an appropriate position in job postings, and learned the skills for completing application materials. They also discussed other issues relevant to job searches such as making arrangements for child care, and identifying appropriate transportation routes, etc. The protocol is available from the authors upon request.

### Information arm

There were two didactic sessions in this arm, each lasting for 2.5 hours. The arm was designed to increase participants' knowledge about Hong Kong's education system, medical care, housing, employment, and community resources in a didactic format.

### Control arm

Participants received a 16-page booklet on knowledge related to Hong Kong's education system, medical care, housing, employment, and community resources.

### Assessments

A significant challenge is to identify existing assessment tools developed in the mainstream of English-speaking Western cultures that fit the intervention content and cultural background of participants (Fabrizio et al., 2012; Stewart et al., 2012). Validated measures are typically long and difficult to comprehend by samples like ours with low education and little experience with questionnaires. Similarly, obtaining observer or behavioral report requires involvement of a third party, and can be regarded as intrusive and unacceptable in this culture. To assess the components we proposed were key to building resilience, we developed our own self-report measures, taking into account the limitations of the context of our participants.

The active components in the Resilience intervention were assessed at baseline and immediately after the intervention. Fourteen items, developed by the research team, were based on the key messages of each session to match the intervention content. The domains were: (1) Self-efficacy, e.g., “find my strengths and virtues” (three items). These items were adapted from measures on self-efficacy and character strengths (Schwarzer and Jerusalem, 1995; Park and Peterson, 2006); (2) Positive thinking, e.g., “adopt positive thinking in the face of adversity” (four items). Similar items were used in previous studies on positive thinking (Kasle et al., 2002; Fredrickson et al., 2003; Stallard and Buck, 2013); (3) Altruism, e.g., “bring my knowledge and skills into full play when helping others” (four items). These items were selected from previous volunteering studies (Mjelde-Mossey and Chi, 2005) and generated based on volunteers' positive experiences (Thoits and Hewitt, 2001; Piliavin and Siegl, 2007); (4) Goal setting, e.g., “set up a clear goal for the next 6 months” (three items). They were designed to match the agency and pathway of hope constructs (Snyder et al., 1991). Exploratory factor analysis showed that the 14 items loaded on four factors corresponding to the content of four Resilience sessions, explaining 69.60% of variance (Table 1). The Cronbach's alpha values for the four domains were all greater than 0.80.

**Table 1:** **Exploratory factor analysis of the four active components**.

**Items**	**Self-efficacy**	**Positive thinking**	**Altruism**	**Goal setting**
1. Appreciate myself	−0.08	−0.08	−0.02	**1.00**
2. Find my strengths and virtues	0.09	0.13	0.11	**0.46**
3. Believe I can	0.19	0.23	0.03	**0.45**
4. Adjust my emotions in the face of setbacks	0.04	**0.78**	−0.13	0.01
5. Adopt positive thinking in the face of adversity	−0.12	**0.77**	0.06	−0.02
6. See difficulties as an opportunity to learn and grow	−0.05	**0.69**	0.02	0.05
7. Use some methods to make myself happier	0.06	**0.62**	0.13	−0.07
8. Enhance my sense of hope in helping others	**0.90**	−0.17	0.07	−0.05
9. Feel that I am fortunate in helping others	**0.82**	−0.03	−0.13	0.10
10. Realize that I can contribute to society in helping others	**0.72**	0.08	0.04	−0.04
11. Bring my knowledge and skills into full play in helping others	**0.51**	0.24	0.04	−0.08
12. Have the motivation to achieve goals	−0.02	−0.02	**0.89**	0.01
13. Set up a clear goal for the next 6 months	−0.04	0.08	**0.77**	0.01
14. Try different methods to achieve goals	0.06	−0.03	**0.69**	0.01

*Factor loadings with absolute value >0.40 are shown in boldface*.

*Extraction Method: Maximum Likelihood*.

*Rotation Method: Promax with Kaiser Normalization*.

Personal resilience was assessed at four time points. The Connor-Davidson Resilience Scale (Connor and Davidson, 2003) was used. Examples of items include “Able to adapt to change” and “Tend to bounce back after illness or hardship.” Test-retest reliability of this scale was 0.87. Convergent validity was shown by significant correlations of the resilience score with hardiness and social support, and discriminant validity was shown by no significant correlation with sexual experience (Connor and Davidson, 2003). The Chinese version has been validated (Yu and Zhang, 2007), and used in a previous study with a different sample to measure personal resilience of immigrants in Hong Kong (Yu et al., 2014c), where it was found to negatively associate as expected with discrimination and rejection, and depressive symptoms. In the present sample, Cronbach's alpha was 0.92 at baseline.

### Statistical analyses

Factor analysis with maximum likelihood and promax rotation was performed to investigate the structure of the 14 items on the four components. Correlations of four components and personal resilience at baseline were conducted with Pearson correlations.

By comparing the Resilience intervention with the Information intervention and Control, we tested the relative effectiveness of the Resilience intervention in changing the four components (potential mediators, Aim 1) from baseline to post-intervention and personal resilience at post-intervention, the 3- and 6-month follow-ups (the outcome measure). The intention-to-treat approach, assuming those with missing follow-up data had no changes from baseline or participants withdrew from the intervention because of lack of benefit (Liu-Seifert et al., 2010), was used. This baseline-observation-carried-forward method is more conservative in handling missing data than last-observation-carried-forward (Scheen et al., 2006), which might over-estimate effect size to favor the hypotheses of effectiveness. Generalized Estimating Equations (GEE) was applied to compare these measures over time in the three arms (Zeger et al., 1988). The effect of the Resilience intervention in changing the four components and personal resilience was indicated by a significant arm by time interaction. Cohen's f was used to indicate effect size (ES), with ≤0.10, 0.25, and ≥0.40 as small, medium, and large effect, respectively (Cohen, 1988).

Mediation tests can elucidate whether a particular construct accounts for change in an outcome following an intervention (Kazdin and Nock, 2003). Mediation models tested that the effect on personal resilience by the Resilience intervention (Resilience vs. Information, and Resilience vs. Control) was mediated by the changes in the four components (Aim 2). We also tested the sustained effects positing that the effect on long term personal resilience by the Resilience intervention was mediated by the changes in short term personal resilience (Aim 3). The MacArthur approach (Kraemer et al., 2002) was used in this study. There are two requirements: (1) temporal relations (Mackinnon, 2008), i.e., that the intervention precedes the change in mediators (e.g., active components at post-intervention), and the mediators change before the outcome (e.g., personal resilience at the 3-month follow-up); (2) association, i.e., the intervention results in the hypothesized mediators, and the proposed mediators are related to the outcome change. Our study design and results fulfilled both criteria. Mediation in linear regression models is indicated by (1) intervention effect on the mediator (a path); and (2) the effect of the mediator on the outcome, adjusted for the intervention, i.e., b path (Mackinnon, 2008). In b path, either a main effect for the mediator or an interaction effect between the intervention and the mediator can indicate the mediator effect on the outcome (Kraemer et al., 2002). Due to the identical results with and without addition of the interaction terms in the current study, we presented the results of main effects without the interaction terms. As the main focus of the present study was sustaining intervention changes, we selected baseline as the reference to control for previous influences of the mediating variables. This approach is common in the literature, for example, Jeffery and French (1999) and Norr et al. (2014). We calculated residualized change scores of the four components from baseline to post-intervention, and tested them in four single-mediator models. Residualized change scores for personal resilience from baseline to post-intervention, from baseline to the 3-month follow-up, and from baseline to the 6-month follow-up as the long term outcome were measured (Tabachnick and Fidell, 2013). Mediation ES was calculated as the ratio of the mediation effect to the standard deviation of dependent variable (Mackinnon, 2008). Confidence interval values were also calculated. All the GEE and mediation results remained identical after demographic characteristics were controlled. For simplicity, we have presented the results without controlling for any covariate. SPSS 21.0 was used for data analyses.

## Results

Demographics and baseline variables of 220 participants are shown in Table 2. The three arms showed no significant differences in demographic variables (gender, age, marital status, educational level, employment status, monthly family income, and duration of settlement after immigration) at baseline (Table 2).

**Table 2:** **Demographics and baseline values of all variables for participants in the three arms**.

	**Information (*n* = 63) n (%) or Mean ± SD (range)**	**Resilience (*n* = 58) n (%) or Mean ± SD (range)**	**Control (*n* = 62) n (%) or Mean ± SD (range)**	***p***
Female	61 (96.8)	55 (94.8)	59 (95.2)	0.85
Age (years)	31.92 ± 4.61 (24–49)	32.97 ± 4.46 (25–43)	33.84 ± 5.56 (22–49)	0.09
Marital status				0.80
Divorced/widowed	2 (3.2)	1 (1.7)	1 (1.6)	
Married	61 (96.8)	57 (98.3)	61 (98.4)	
Education level				0.21
Primary education	5 (7.9)	8 (13.8)	7 (11.3)	
Secondary education	43 (68.3)	45 (77.6)	46 (74.2)	
Technical/high school/tertiary education	15 (23.8)	5 (8.6)	9 (14.5)	
Employment status				0.07
Housewife/unemployed	56 (88.9)	56 (96.6)	52 (83.9)	
Employed	7 (11.1)	2 (3.4)	10 (16.1)	
Monthly family income (HK$1)				0.89
≤9999	26 (41.3)	29 (50.0)	29 (46.8)	
10,000–14,999	24 (38.1)	19 (32.8)	20 (32.3)	
≥15,000	13 (20.6)	10 (17.2)	13 (21.0)	
Duration of settlement after immigration				0.47
≤1 month	23 (36.5)	19 (32.8)	20 (32.3)	
2–6 months	22 (34.9)	28 (48.3)	30 (48.4)	
≥7 months	18 (28.6)	11 (19.0)	12 (19.4)	
Adaptation difficulties	71.18 ± 15.07 (41–98)	75.02 ± 16.91 (32–111)	64.74 ± 19.19 (28–107)	0.01
Knowledge	11.87 ± 4.27 (3–21)	11.50 ± 4.86 (1–24)	11.79 ± 4.55 (1–22)	0.90
Personal resilience	57.73 ± 14.58 (23–93)	58.23 ± 12.16 (32–91)	59.32 ± 16.35 (21–96)	0.82
Depressive symptoms	5.03 ± 4.22 (0–20)	4.71 ± 3.84 (0–14)	4.61 ± 3.53 (0–12)	0.82

*US$1 = HK$7.80*.

*All scores were computed so that higher values indicate higher levels of the variables named. For example, higher scores in adaptation difficulties indicate more adaptation difficulties, and higher scores in personal resilience indicate more personal resilience*.

The four components were created to correspond to the foci of our four sessions. As would be expected for contributors to the same umbrella construct of resilience, they were moderately intercorrelated (*r*'s ranging from 0.42 to 0.65, Table 3). All the four components were positively associated with personal resilience at baseline (correlation coefficients ranged from 0.42 to 0.58, all *p* < 0.001, Table 3).

**Table 3:** **Pearson correlations of four active components and personal resilience at baseline**.

	**Positive thinking (*p*)**	**Altruism (*p*)**	**Goal setting (*p*)**	**Personal resilience (*p*)**
Self-efficacy	0.65 (<0.001)	0.51 (<0.001)	0.56 (<0.001)	0.58 (<0.001)
Positive thinking	–	0.42 (<0.001)	0.56 (<0.001)	0.58 (<0.001)
Altruism	–	–	0.54 (<0.001)	0.42 (<0.001)
Goal setting	–	–	–	0.55 (<0.001)

### Effectiveness of the resilience intervention in changing the four active components from baseline to post-intervention

**Figure 3:**
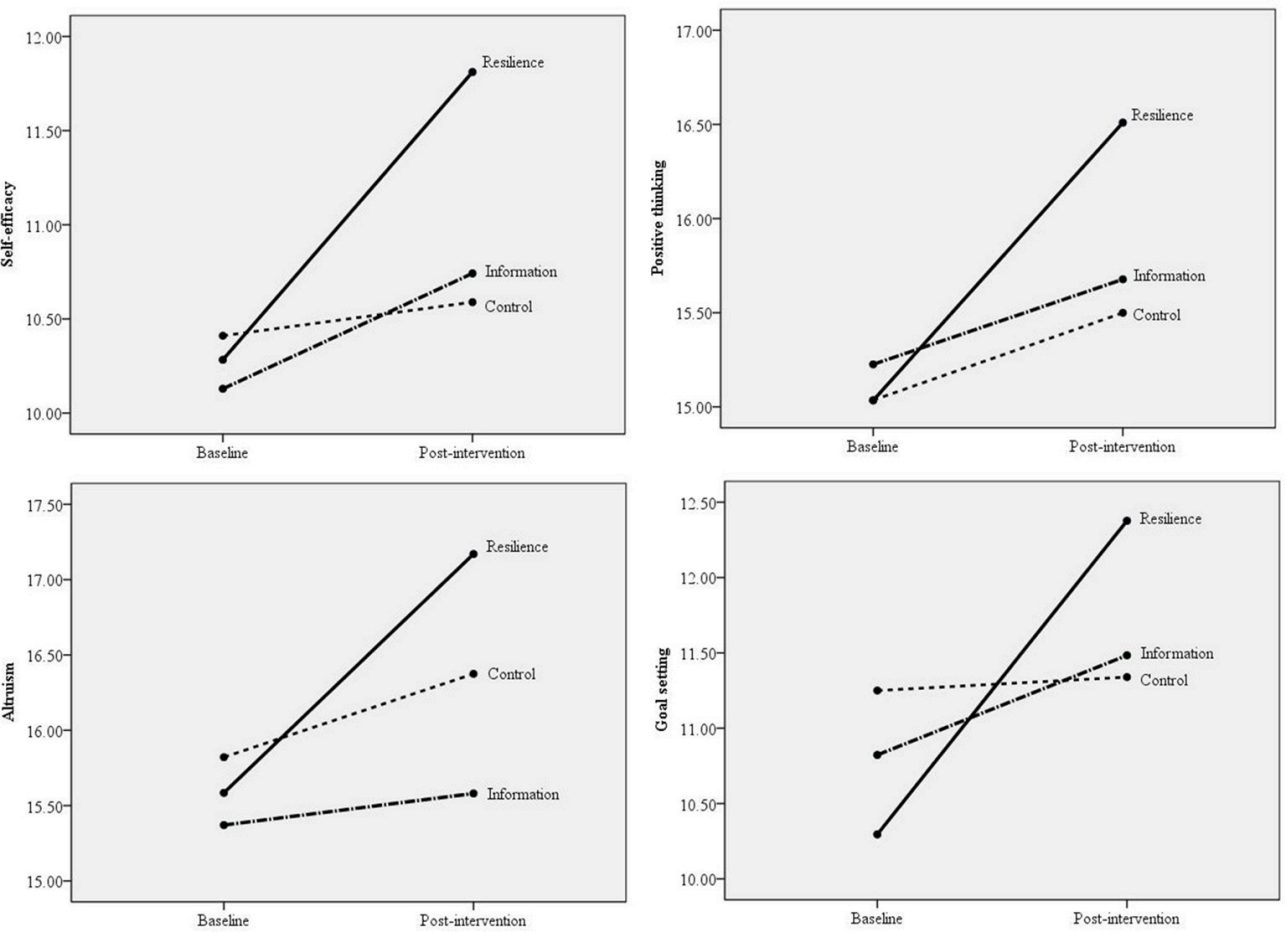
**Comparisons of three arms in changes in the four active components (self-efficacy, positive thinking, altruism, and goal setting) from baseline to post-intervention**.

GEE results in Table 4 showed that participants in the Resilience arm reported greater post-intervention increases in the four active components than those in the Information arm (all *p* < 0.05, ES ranged from 0.22 to 0.29, around medium level) and the Control arm (all *p* < 0.05, ES ranged from 0.19 to 0.47, small to large levels; Figure 3).

**Table 4:** **Effectiveness analysis for comparisons of three arms in Generalized Estimating Equations models**.

	**Resilience vs. Information**			**Resilience vs. Control**		
	**Wald χ^2^**	***p***	**Effect size**	**Wald χ^2^**	***p***	**Effect size**
**SELF-EFFICACY**						
Baseline to post-intervention						
Arm effect	5.61	0.02		3.89	0.048	
Time effect	35.80	<0.001		20.89	<0.001	
Arm × time effect	6.02	0.01	0.22	12.63	<0.001	0.32
**POSITIVE THINKING**						
Baseline to post-intervention						
Arm effect	0.79	0.37		1.89	0.17	
Time effect	20.67	<0.001		21.70	<0.001	
Arm × time effect	5.94	0.02	0.24	5.13	0.02	0.22
**ALTRUISM**						
Baseline to post-intervention						
Arm effect	5.28	0.02		1.68	0.20	
Time effect	15.23	<0.001		27.97	<0.001	
Arm × time effect	9.27	0.002	0.29	4.78	0.03	0.19
**GOAL SETTING**						
Baseline to post-intervention						
Arm effect	0.30	0.58		0.19	0.66	
Time effect	46.40	<0.001		36.80	<0.001	
Arm × time effect	12.50	<0.001	0.26	27.74	<0.001	0.47
**PERSONAL RESILIENCE**						
Baseline to post-intervention						
Arm effect	0.39	0.53		0.17	0.68	
Time effect	10.13	0.001		4.85	0.03	
Arm × time effect	0.86	0.35	0.09	5.33	0.02	0.20
Baseline to 3-month follow-up						
Arm effect	1.77	0.18		0.56	0.46	
Time effect	10.72	0.01		5.95	0.05	
Arm × time effect	5.96	0.05	0.23	5.78	0.06	0.20
Baseline to 6-month follow-up						
Arm effect	2.28	0.13		0.42	0.52	
Time effect	11.37	0.01		6.20	0.10	
Arm × time effect	6.64	0.08	0.23	6.79	0.08	0.22

*Analyses were based on Generalized Estimating Equations*.

*Effect sizes are indicated as Cohen's f (1988), and defined as small ≤0.10, medium = 0.25, and large ≥0.40*.

### Effectiveness of the resilience intervention in changing personal resilience at post-intervention and the two follow-ups

**Figure 4:**
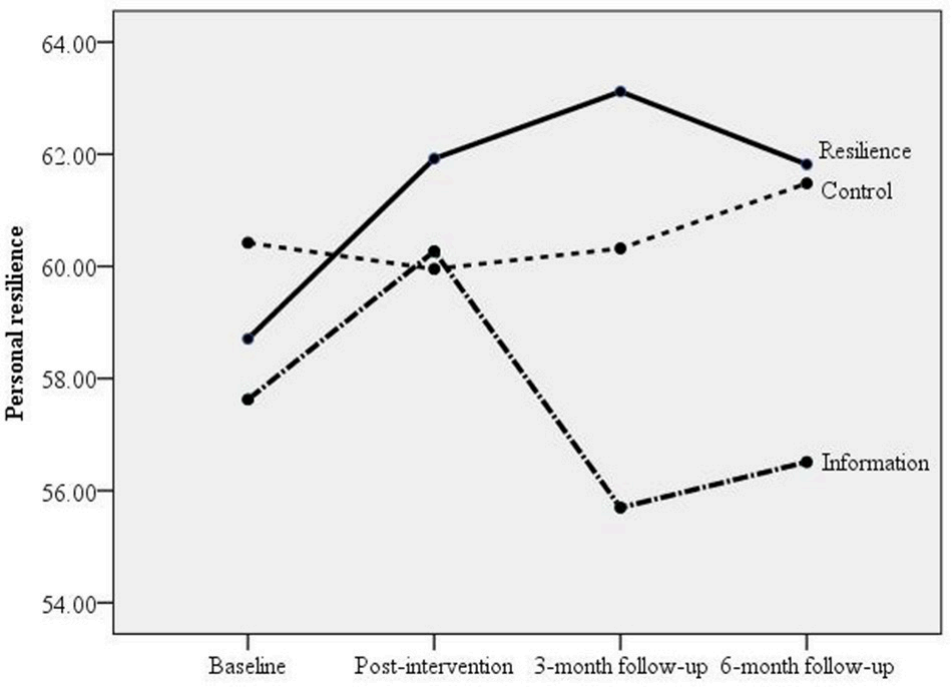
**Comparisons of three arms in changes in personal resilience from baseline to post-intervention, 3-month and 6-month follow-ups**.

GEE results in Table 4 showed that compared with participants in the Information arm, those in the Resilience arm reported marginally greater increases in personal resilience at the 3-month (*p* = 0.05, ES = 0.23, close to medium level) and 6-month follow-ups (*p* = 0.08, ES = 0.23, close to medium level). Compared with participants in the Control arm, those in the Resilience arm reported greater increases in personal resilience post-intervention (*p* = 0.02, ES = 0.20, small level) and marginally significant increases at the 3-month (*p* = 0.06, ES = 0.20, small level) and 6-month follow-ups (*p* = 0.08, ES = 0.22, close to medium level; Figure 4).

### Mediation effects of the four active components on the short term outcome of personal resilience

**Figure 5:**
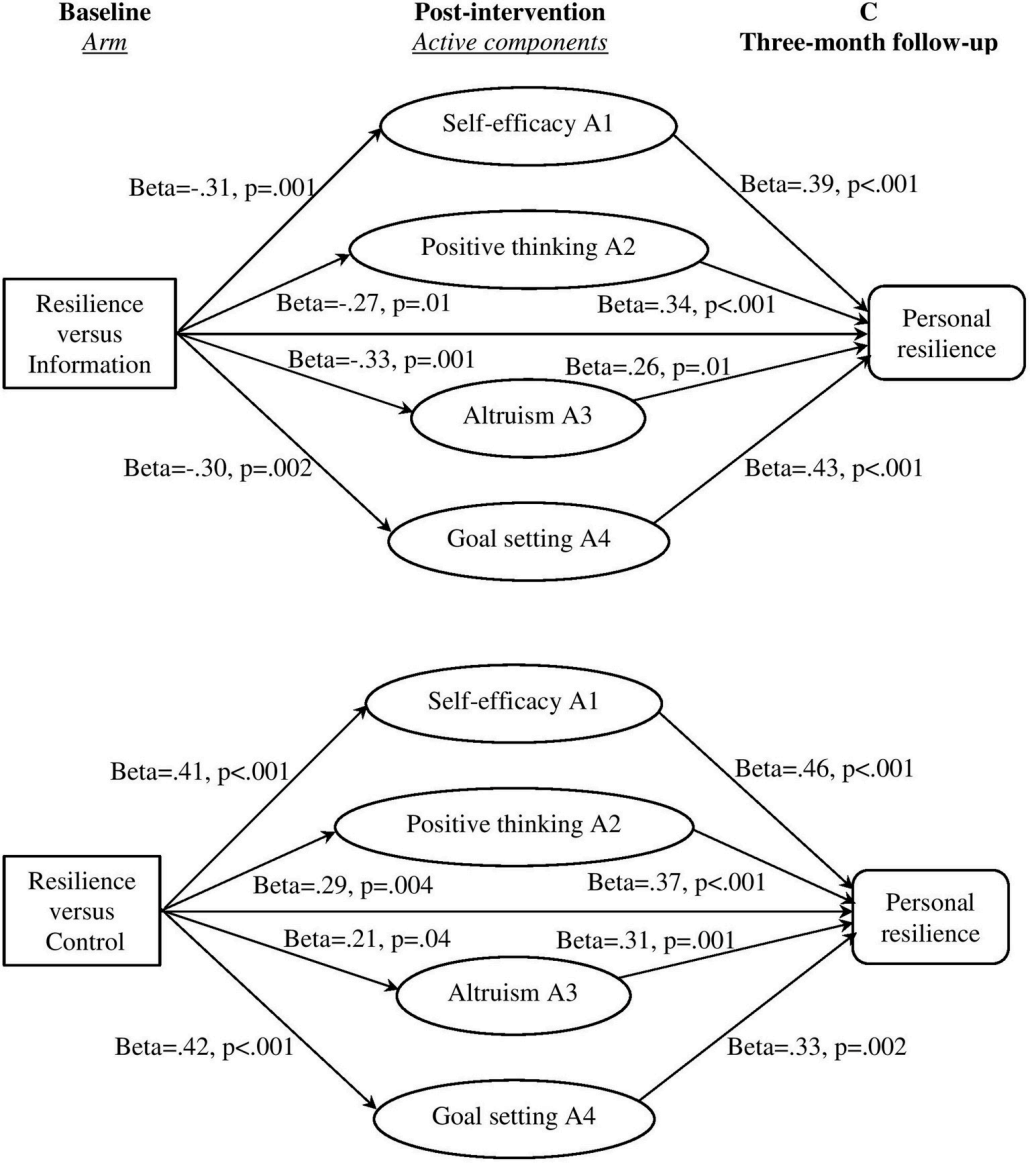
**Mediation effects of the four active components from baseline to post-intervention on the outcome of personal resilience at the 3-month follow-up**.

Our data fulfilled the second criterion regarding association in the MacArthur approach, as the Resilience intervention resulted in the proposed mediators (see GEE results above, Table 4), and these mediators were correlated with outcome changes (see partial correlation results after controlling for arm effects, Tables 5, 6). In mediation analyses for the comparison of the Resilience vs. Information arms, there were significant mediator effects of the four components (A) on the outcome of personal resilience at the 3-month follow-up (C) (all *p* values for a and b paths < 0.05, Table 7). The mediation ES for self-efficacy (A1), positive thinking (A2), altruism (A3), and goal setting (A4) were −0.24 (95% CI: −0.41, −0.09), −0.18 (95% CI: −0.35, −0.05), −0.17 (95% CI: −0.41, −0.002), and −0.25 (95% CI: −0.43, −0.09), respectively (Figure 5).

**Table 5:** **Partial correlations between changes of four active components from baseline to post-intervention and changes of personal resilience from baseline to 3-month follow-up**.

	**Resilience vs. Information**	**Resilience vs. Control**
Self-efficacy (A1)	0.27 (0.01)	0.35 (<0.001)
Positive thinking (A2)	0.24 (0.01)	0.26 (0.01)
Altruism (A3)	0.19 (0.05)	0.27 (0.01)
Goal setting (A4)	0.35 (<0.001)	0.23 (0.02)

**Table 6:** **Partial correlations among changes of personal resilience at different time points**.

	**Resilience vs. Information**		**Resilience vs. Control**	
	**1**	**2**	**1**	**2**
Personal resilience from baseline to post-intervention	–		–	
Personal resilience from baseline to the 3-month follow-up	0.51 (<0.001)	–	0.48 (<0.001)	–
Personal resilience from baseline to the 6-month follow-up	0.55 (<0.001)	0.68 (<0.001)	0.50 (<0.001)	0.65 (<0.001)

**Table 7:** **Effects of residualized change score in four active components from baseline to post-intervention (A) as a mediator of arm effect on the outcome of residualized change score of personal resilience measured 3 months after the interventions (C)**.

	**Resilience vs. Information**				**Resilience vs. Control**			
	**Beta**	***t***	***p***	**Adjusted R^2^**	**Beta**	***t***	***p***	**Adjusted R^2^**
**SELF-EFFICACY (A1)**								
Intervention effect on the mediator (a path)				0.09				0.16
Arm (a)	−0.31	−3.35	0.001		0.41	4.47	<0.001	
Mediator effect on the outcome (c' and b paths)				0.17				0.20
Arm (c')	−0.11	−1.15	0.26		0.0001	−0.001	0.99	
Mediator main effect (b)	0.39	4.23	<0.001		0.46	4.75	<0.001	
**POSITIVE THINKING (A2)**								
Intervention effect on the mediator (a path)				0.06				0.07
Arm (a)	−0.27	−2.84	0.01		0.29	2.98	0.004	
Mediator effect on the outcome (c' and b paths)				0.14				0.14
Arm (c')	−0.13	−1.43	0.16		0.08	0.87	0.39	
Mediator main effect (b)	0.34	3.68	<0.001		0.37	3.83	<0.001	
**ALTRUISM (A3)**								
Intervention effect on the mediator (a path)				0.10				0.03
Arm (a)	−0.33	−3.59	0.001		0.21	2.11	0.04	
Mediator effect on the outcome (c' and b paths)				0.10				0.11
Arm (c')	−0.14	−1.44	0.15		0.12	1.29	0.20	
Mediator main effect (b)	0.26	2.70	0.01		0.31	3.27	0.001	
**GOAL SETTING (A4)**								
Intervention effect on the mediator (a path)				0.08				0.17
Arm (a)	−0.30	−3.16	0.002		0.42	4.63	<0.001	
Mediator effect on the outcome (c' and b paths)				0.21				0.11
Arm (c')	−0.10	−1.12	0.26		0.05	0.50	0.62	
Mediator main effect (b)	0.43	4.78	<0.001		0.33	3.20	0.002	

For the comparison of the Resilience vs. Control arms, all the four components (A) had significant mediation effects on the 3-month follow-up personal resilience (C) (all *p*-values for a and b paths < 0.05, Table 7). The mediation ES for self-efficacy (A1), positive thinking (A2), altruism (A3), and goal setting (A4) were 0.37 (95% CI: 0.20, 0.57), 0.21 (95% CI: 0.06, 0.39), 0.13 (95% CI: 0.01, 0.30), and 0.28 (95% CI: 0.11, 0.47), respectively (Figure 5).

### Mediation effects of the short term outcomes of personal resilience on the long term outcome of personal resilience

**Figure 6:**
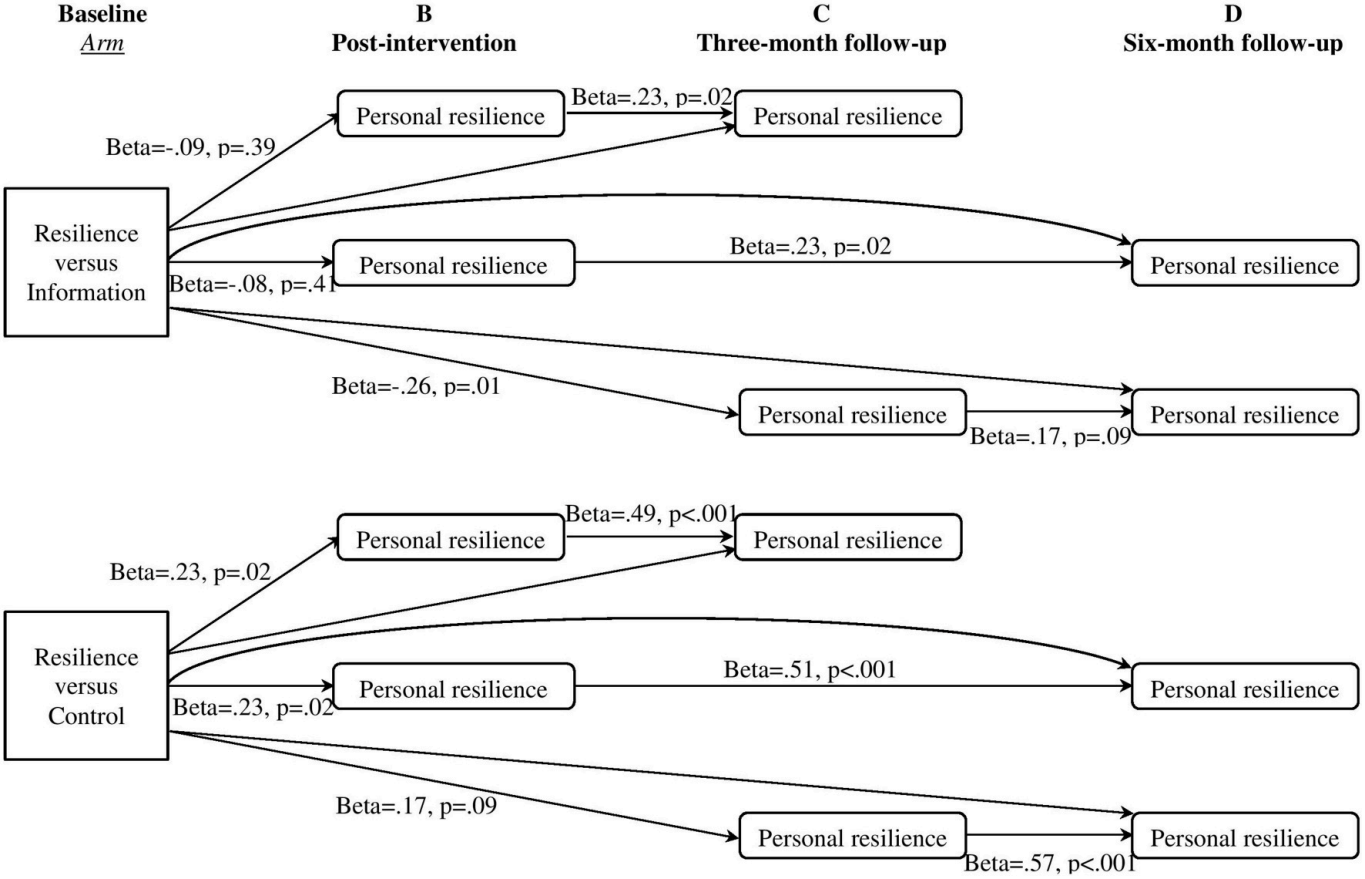
**Mediation effects of short-term personal resilience on the outcome of longer-term personal resilience**.

Table 8 shows that, for the comparison of the Resilience vs. Information arms, there was no significant intervention effect on the mediator (a path) in the mediation tests of personal resilience from baseline to post-intervention (B) on the outcome of change in personal resilience at the 3-month follow-up (C) (mediation ES = −0.09; 95% CI: −0.32, 0.11) and the 6-month follow-up (D) (mediation ES = −0.09; 95% CI: −0.30, 0.12). The mediation effect of personal resilience at the 3-month follow-up (C) was significant for the outcome of change of personal resilience at the 6-month follow-up (D), and mediation ES was −0.33 (95% CI: −0.58, −0.09). The mediation effects were depicted in Figure 6.

**Table 8:** **Effects of residualized change score in short term personal resilience as a mediator of arm effect on the outcome of residualized change score of longer term personal resilience**.

	**Resilience vs. Information**				**Resilience vs. Control**			
	**Beta**	***t***	***p***	**Adjusted R^2^**	**Beta**	***t***	***p***	**Adjusted R^2^**
**IV = Arm**								
**Mediator = Personal resilience from baseline to post-intervention (B)**								
**DV = Personal resilience from baseline to the 3-month follow-up (C)**								
Intervention effect on the mediator (a path)				0.002				0.04
Arm (a)	−0.09	−0.87	0.39		0.23	2.31	0.02	
Mediator effect on the outcome (c' and b paths)				0.32				0.25
Arm (c')	−0.18	−2.21	0.03		0.07	0.82	0.42	
Mediator main effect (b)	0.53	6.57	<0.001		0.49	5.56	<0.001	
**IV = Arm**								
**Mediator = Personal resilience from baseline to post-intervention (B)**								
**DV = Personal resilience from baseline to the 6-month follow-up (D)**								
Intervention effect on the mediator (a path)				0.003				0.04
Arm (a)	−0.08	−0.83	0.41		0.23	2.36	0.02	
Mediator effect on the outcome (c' and b paths)				0.32				0.23
Arm (c')	−0.17	−2.07	0.04		−0.05	−0.52	0.60	
Mediator main effect (b)	0.54	6.77	<0.001		0.51	5.65	<0.001	
**IV = Arm**								
**Mediator = Personal resilience from baseline to the 3-month follow-up (C)**								
**DV = Personal resilience from baseline to the 6-month follow-up (D)**								
Intervention effect on the mediator (a path)				0.06				0.02
Arm (a)	−0.26	−2.71	0.01		0.17	1.74	0.09	
Mediator effect on the outcome (c' and b paths)				0.43				0.31
Arm (c')	−0.05	−0.66	0.51		−0.03	−0.37	0.71	
Mediator main effect (b)	0.65	8.64	<0.001		0.57	6.85	<0.001	

For the comparison of the Resilience vs. Control arms, there were significant mediation effects of personal resilience from baseline to post-intervention (B) on the outcome of change of personal resilience at the 3-month follow-up (C) (mediation ES = 0.22; 95% CI: 0.03, 0.49) and the 6-month follow-up (D) (mediation ES = 0.23; 95% CI: 0.04, 0.46). However, in the mediation test of personal resilience at the 3-month follow-up (C) on the outcome of change of personal resilience at the 6-month follow-up (D), the intervention effect on the mediator (a path) was not significant (*p* < 0.10, at trend level; mediation ES = 0.19; 95% CI: −0.01, 0.42; Figure 6).

## Discussion

Our study examined the mediators in an intervention program to develop culturally-acceptable preventive programs for an immigrant sample with low income and education, outside the West. We hope that it demonstrates a step toward the challenging goal of improvements in the science of preventive mental health interventions in culturally diverse groups. Supplementing previous results that our Resilience intervention improved short term personal resilience (Yu et al., 2014b), the findings in the present report shows that the intervention also effectively enhanced the four active components. By separately testing each component in the multi-component intervention, we established that the four components were all separately responsible for personal resilience enhancement 3 months following the completion of intervention. Self-efficacy and goal setting emerged as having greater impact on personal resilience compared with positive thinking and altruism. In addition, follow-up analyses found that the Resilience intervention significantly enhanced personal resilience 6 months following the completion of the intervention, and short term personal resilience enhancement predicted longer term personal resilience enhancement. These findings inform the mechanisms of intervention, addressing a gap in intervention studies which are increasingly recognized but not well established statistically (Kazdin, 2007).

It is not surprising to find that by comparing their contributions in the mediation models, self-efficacy and goal setting played the largest roles in mediating the intervention effect on personal resilience at the 3-month follow-up than the other two components of positive thinking and altruism. Self-efficacy, the belief that a person has ability to perform a task (Bandura, 1982), has a central function as its relevance to the umbrella concept of resilience (Rutter, 1987; Bradshaw et al., 2007). People with high levels of self-efficacy are more likely to be optimistic, hopeful, and skillful in mobilizing coping resources to overcome difficulties and adversities. This element can maximize the individual's ability to take care of himself or herself and people around him or her (Benedek et al., 2007). Self-efficacy has been widely used in mental health promotion intervention in different cultural contexts for various populations (Mueller et al., 2011; Boldor et al., 2012; Barry et al., 2013), but few studies have empirically tested whether the alteration effected as a result of the intervention explains the variability in the intervention outcome. In addition, goal setting, an integral manifestation of resilience, comprises of positive transformation in new opportunities and focuses on enactment of hope about a better future (Stajduhar et al., 2009). This component is expected to enhance persistence and commitment to pursuing desirable outcomes, even though obstacles may be encountered (Martin and Marsh, 2006). Goal setting also increases participants' resilience by strengthening their feeling of control (Collins, 2007), enhancing their assets, improving risk-management skills, and facilitating effective adaptation (Youssef and Luthans, 2007). A task of goal setting in our session required participants to set up goals in the coming 6 months; this kind of future orientation to motivate actions may have more sustained effects on personal resilience enhancement (Seginer, 2008) compared to other resilience components such as positive thinking and altruism whose effects may be depleted quickly following the intervention.

Our design limited the interpretations of the findings regarding relative changes of the mediators in relation to the intervention. Specifically, we did not test each mediator at every step, and thus could not determine that the change was exclusively the result of the module that targeted it. Nor could we say that the full specific effects of each module on the mediator were evident. For example, the modules might have contributed to mediators that were not directly targeted, and/or the effect on a non-targeted mediator might have been present soon after the module was delivered but was not sustained. However, these findings have some implications for future generations of the intervention. Efforts to ratchet up the effects of the targeted mediated processes that showed less change might be a goal. Future studies might determine the individual effectiveness of each “module,” with the goal to develop a library of single-session interventions that might be combined in various ways depending upon the need, or utilized to supplement other interventions aiming at relevant outcomes. Empirical tests of whether the two “largest” contributors targeted in a briefer intervention could be relevant to further reducing the number of sessions which is an important goal in public health. Adaptation to new settings might be guided by the mediator—altruism may take different forms in different age groups and cultures. For example, an intervention to enhance resilience via altruism in an acutely ill child may consist of helpful acts toward siblings, parents, classmates, or nursing staff.

A few of the intervention effects on personal resilience were weak, indicating that the resilience intervention needs to be enhanced for a stronger impact. However, five of six comparisons yielded effect sizes close to moderate. Small effect sizes matter in this population as they are struggling to adapt to a new environment, and even minor improvements might boost their confidence. From a public health point of view, small effects from low cost interventions spread over a large population can make a significant difference in the health of the community. These findings provide initial evidence in developing science for intervention studies in culturally diverse settings. In some tests of sustained effects, non-significant intervention effects on mediators (a path) suggest that a more powerful resilience intervention is needed to change the mediators of personal resilience that were targeted at post-intervention and the 3-month follow-up. An important finding was that the intervention impact on longer term outcome of personal resilience was predicted by short term changes in personal resilience in some models. This finding highlights the sustained intervention effects. This result also strongly emphasizes the need for multiple assessments following the completion of intervention programs in evaluation design (Kazdin, 2007), which allows the early outcome to be measured and tested as the best predictor for later improvement. An implication of this model for resilience research is that it is crucial to leverage improvement in outcomes even by a small amount immediately following the intervention, as this seems to consolidate characteristics and resources that predict longer term maintenance of changes.

These analyses are a single step in the analyses of the mechanisms of effect. Identifying the critical agents by which change is achieved by using mediation tests is a weak demonstration of causality (Murphy et al., 2009). Dismantling the intervention package to examine the causal relationships is the next step (Fortier et al., 2011). Dismantling efforts for an intervention like ours might include manipulating self-efficacy to achieve low, medium, and high levels in different subgroups, testing equivalent changes in outcomes (Kazdin, 2007), and demonstrating that no change occurs without this component (Lerner et al., 2012).

The intervention effect at post-intervention on personal resilience was not significant when the Resilience arm was compared with the Information arm. This finding makes sense, as the Information arm was also effective in improving adaptation of immigrants as expected and indicated in our previous report (Yu et al., 2014b). The reader may attribute this to the Dodo bird effect, which claims that all psychotherapies, regardless of the specific components, generate equivalent outcomes, and common factors that are shared in different interventions (e.g., warm therapeutic relationships, positive expectations) may result in improvements (Marcus et al., 2014). However, the Resilience intervention did surpass the Information intervention in the follow-up period, and our mediation results showed that the four active components and personal resilience at earlier stages contributed to the observed improvements in personal resilience at a later time. Direct comparisons of the Resilience intervention against another active intervention that has been shown to be effective in improving resilience would contribute to disentangling the Dodo bird effect (Benish et al., 2008).

There are several limitations in the present study. First, women were over-represented among our participants as they constitute the majority of immigrants from mainland to Hong Kong. It would be helpful to explore differences in mediation of adaptation in an immigrant population that included more men. Second, we used self-report to assess personal resilience, which may not accurately reflect the behavioral ability to persist in the face of adversity. This possibility is somewhat mitigated by the fact that our primary outcome for the intervention was adaptation difficulties and self-reported resilience was a mediator for change in that outcome (Yu et al., 2014b). Adjustment to a new environment requires the ability to endure stressful events and persevere with goals in the face of adversity. Furthermore, self-reported resilience measured by the Connor-Davidson Resilience Scale has been associated with various adjustment outcomes, including sustained engagement with the tasks (Campbell-Sills et al., 2006). This measure has also been shown to successfully indicate improvement following psychotherapy and psychological interventions (Davidson et al., 2008; Sood et al., 2011). Nevertheless, future studies should include a behavioral and/or observational assessment of resilience. Third, our measures of the four mediators were “home-grown,” developed specifically for this program based on a review of the literature and input from the community. It is possible that they measured some general underlying factor such as well-being or positive self-image, rather than the specific constructs we had targeted. They would have been stronger from a psychometric point of view had we included methods that would allow tests of discriminant validity. We relied on brief measures developed for this study to assess the four components. Thus, the psychometric properties of the instruments were not previously demonstrated in an independent study. These components were designed to capture the intervention block content, so there was a possibility that they overestimated intervention effects. Although, we assessed adaptation difficulties in our previous report, no external assessments such as social or cultural competencies were included in the assessment. There is a need to develop measures in non-Western and undereducated samples in culturally diverse settings. In addition, we measured self-efficacy entirely through self-report, consistent with its conceptualization as a belief. Beliefs should translate into mood and behavioral changes, and it could further the comprehensive measurement base of this construct to consider additional observable measures that reflect self-efficacy in future studies. Fourth, the four components were measured only at pre- and post-intervention, and no information was available about these changes after every session. An intensive time series approach to assess these components repeatedly, e.g., during the intervention, would have provided an opportunity for a more thorough investigation of mediation. In an ideal study, mediators and outcomes would be measured frequently and simultaneously at multiple time points in order to discern the time sequence and determine whether the change in mediators precede the change in outcomes or vice versa. However, “assessment fatigue” has been noted in other samples (Bausewein et al., 2010). This pitfall is of particular concern in culturally diverse individuals with a low level of formal education and low tolerance of repetitive written materials. Further research is needed to address the challenge of boredom and burden resulting from repeated measures in participants who are not familiar with self-report questionnaires. In addition, no information on test-retest and treatment sensitivity for the measures was available. Fifth, participants and facilitators were not blind to the treatment condition. In contrast to pharmacotherapy trials, it is notoriously difficult and frequently impossible to implement blinding in psychological interventions (Schulz and Grimes, 2002; Baumeister et al., 2014). The facilitators who delivered the interventions were aware of the intervention content and techniques, and the participants expected improvements in specific outcomes based on the material covered. It would be difficult to manipulate the facilitators and participants into the belief that the Resilience condition would lead to increases in knowledge. However, two mitigating issues might be considered. The first is that all participants and interventionists expected that the intervention arm in which they participated would result in improved adaptation. Second, this study examined the links between specific strategies and the pathways to that improvement. The intervention focus was on adaptation, so expectations of improvement were not obviously discrepant in the different groups. Non-specific treatment effects were not controlled or tested in the present study (Newton-John and Geddes, 2008). Including objective measures in future studies might be helpful to reduce the potential bias when it is difficult to implement blinding procedures. Furthermore, the Resilience intervention included more contact time. This factor might account for the differences in effects to the Information and control conditions, but the impact was unlikely to be substantial. The design was driven by our interest in the building blocks of an eventual multi-targeted intervention (i.e., Resilience plus Information interventions as a comprehensive package). Thus, we wanted to make an impact on each block most efficiently. We acknowledge that this difference may have influenced the effectiveness results. Future studies are needed to test the Resilience intervention against other effective psychosocial interventions with the same treatment duration. Consistent with recent discussions about sudden changes in the discontinuous trajectories of improvement before the intervention started (Heinzel et al., 2014), there may be other explanations for the intervention effects. Trans-information measures or time series data might be effective strategies for future studies to exclude such effects. Sixth, although we demonstrated that four active components and personal resilience at earlier stages mediated the intervention effects on personal resilience, the possibility that common factors (e.g., positive expectations, peer support) worked to improve the effects cannot be conclusively excluded. Future studies might consider examining the effects of common factors on the outcomes. Finally, sample size estimation was driven by the goal to increase personal resilience. It may not have been adequate for mediation analysis. The small sample size in the present study may account for the non-significant 95% CI values in the three mediation tests on sustained effects.

In this randomized controlled trial, we presented an explanation about intervention mechanisms with regard to the core and active components that mediated personal resilience enhancement. Our study contributes to the literature as it demonstrated a mediation model, with change in targeted variables associated with change in a proximal outcome, which then is associated with changes in a more distal outcome. We also identified possible “building blocks” in a multiple-session intervention program that could be adapted and individually selected or combined. In addition, our findings partially supported the sustained effects that short term personal resilience changes predicted subsequent longer term personal resilience changes. The evidence of these two independent and theoretically consistent mechanisms of change may reflect two different paths toward resilience enhancement over the shorter compared to the longer term. Such evidence provides further understanding about theoretical models of resilience enhancement, and informs development, improvement, and generalization of evidence-based psycho-social interventions. Although, there are a few methodological limitations including using self-developed subjective measures to assess active components, this study captured the key components for resilience enhancement that are acceptable to a culturally diverse sample and paved the way for future program improvements in non-Western cultures. In future studies, the Resilience intervention needs modification to produce stronger intervention effects. Further evidence is needed to test the brief Resilience intervention against other proven-effective psychosocial interventions. For study design, an intensive time series approach is recommended to assess the active components together with common factor dynamics during the intervention. Our findings suggest that in addition to designing specific strategies and policies, targeting these active components for resilience enhancement in immigrants, creating opportunities in the natural environment to cultivate these components (e.g., increasing self-efficacy in parenting, promoting positive thinking in school applications of kids, setting feasible goals in financial management) might be helpful. As this preventive program was designed to target a wide range of immigrants, social services based on these strategies can be offered across the board to all immigrants from Mainland China to Hong Kong.

## Authors contributions

NY was responsible for overall coordination, implementation, data collection and analysis, result interpretation, and manuscript writing. TL contributed to study design, implementation, result interpretation, and manuscript writing. IL monitored field work. SS worked on the interpretation and formulation of the findings.

### Conflict of interest statement

The authors declare that the research was conducted in the absence of any commercial or financial relationships that could be construed as a potential conflict of interest.
